# THE POWER OF ARTS-BASED ACTIVITIES: APPLICATIONS OF AN INTERGENERATIONAL HAIKU-MAKING ACTIVITY

**DOI:** 10.1093/geroni/igae098.0467

**Published:** 2024-12-31

**Authors:** Yoshiko Matsumoto, Harumi Maeda

**Affiliations:** Stanford University, Stanford, California, United States; Stanford University, Stanford, California, United States

## Abstract

The power of arts- and humanities-based activities for enhancing the well-being and quality of life of older adults is well documented in the existing literature (e.g., Fraser et al., 2015; Letrondo et al., 2023; Young et al., 2016). An intergenerational haiku-making activity designed and implemented by the authors et. al. (2021, 2024) is one such activity. In this activity, 2-3 older adults living with mild cognitive impairment (MCI) interact with 2-3 university students to create haiku poems based on seasonal photos of their choice, followed by short one-on-one feedback interviews. Analysis of the recorded activity sessions demonstrates how the activity created meaningful outcomes for both older and younger participants, including increased emotional well-being, intergenerational understanding, and self-expression. This presentation discusses two applications of the intergenerational haiku-making activity in two different settings. First, we designed and taught a community-engaged learning course in which undergraduate and graduate students learned first-hand how humanities-based intergenerational activities enhance intergenerational communication and mutual understanding. Second, the activity was re-designed to target older Japanese immigrants living with cognitive impairment by incorporating elements that encourage them to draw upon their Japanese language heritage. The bilingual version will be implemented in a local Japan-focused adult day services program. This presentation highlights the effectiveness of arts- and humanities-based activities by showing the ways in which one particular activity can be expanded and applied in different settings to offer tailored benefits to people of varying ages and cognitive conditions.

